# Who chooses “healthy” meals? An analysis of lunchtime meal quality in a workplace cafeteria

**DOI:** 10.1186/s12889-024-18284-5

**Published:** 2024-03-29

**Authors:** Sally L. Bullock, Hilary M. Winthrop, Derek Hales, Feng-Chang Lin, Yumei Yang, Alice S. Ammerman, Anthony J. Viera

**Affiliations:** 1 Davidson College, PO Box 5000, 209 Ridge Road, Davidson, NC 28035 USA; 2 Duke University School of Medicine, 2200 W Main St, Durham, NC 27705 USA; 3 Center for Health Promotion and Disease Prevention, University of North Carolina at Chapel Hill, 1700 MLK Jr. Blvd, CB#7426,, Chapel Hill, NC 27599 USA; 4 Department of Biostatistics, Gillings School of Global Public Health, University of North Carolina at Chapel Hill, 135 Dauer Drive, 3101 McGavran-Greenberg Hall, CB #7420,, Chapel Hill, NC 27599 USA; 5 Department of Nutrition, Gillings School of Global Public Health, Center for Health Promotion and Disease Prevention, University of North Carolina at Chapel Hill, CB# 7426, 1700 MLK Jr. Blvd, Room 239,, Chapel Hill, 27599 USA; 6 Department of Family Medicine and Community Health, Duke University School of Medicine, 2200 W Main St, Suite 400, Durham, NC 27705 USA

**Keywords:** Meal quality, Diet index, Nutrition assessment, Nutrition surveys, Worksite

## Abstract

**Background:**

The workplace can play an important role in shaping the eating behaviors of U.S. adults. Unfortunately, foods obtained in the workplace tend to be low in nutritional quality. Questions remain about the best way to approach the promotion of healthy food purchases among employees and to what extent health promotion activities should be tailored to the demographic characteristics of the employees. The purpose of this study was to (1) assess the nutritional quality of lunchtime meal purchases by employees in cafeterias of a large organization, (2) examine associations between lunchtime meal quality selection and the demographic characteristics of employees, and (3) determine the healthfulness of foods and beverages offered in the cafeterias of this organization.

**Methods:**

A cross-sectional analysis was conducted using secondary data from a food labeling study implemented in three worksite cafeterias. Demographic data was collected via surveys and meal data was collected using a photo capture system for 378 participants. The Healthy Eating Index 2015 (HEI-2015) was used to determine meal quality and a total score for the menu of options available in the cafeterias during the study period. Summary statistics were generated, and the analysis of variance (ANOVA) was used to compare the HEI-2015 scores between groups.

**Results:**

The mean HEI-2015 total score for the menu items offered (*n* = 1,229) in the cafeteria during the study period was 63.1 (SD = 1.83). The mean HEI-2015 score for individual lunchtime meal observations (*n* = 378) was 47.1 (SD = 6.8). In general, HEI-2015 total scores were higher for non-smokers, individuals who self-identified as Asian, had higher physical activity levels, scored higher on numeracy and literacy assessments, and reported higher education levels, incomes, and health status.

**Conclusions:**

The overall HEI-2015 scores indicate that the menu of options offered in the cafeterias and individual meal selections did not align with the Dietary Guidelines for Americans, and there were significant associations between average lunchtime meal quality scores and several demographic characteristics. These results suggest that healthy eating promotion activities in workplaces may need to be tailored to the demographic characteristics of the employees, and efforts to improve the food environment in the workplace could improve meal quality for all employees.

**Supplementary Information:**

The online version contains supplementary material available at 10.1186/s12889-024-18284-5.

## Background

Unhealthy diets contribute to the development of non-communicable diseases, including cardiovascular disease, type 2 diabetes, and some types of cancer [1, 2], and may also contribute to worse health outcomes from COVID-19 [3]. While there have been some improvements in the dietary quality of Americans over the last several decades, many Americans have suboptimal diets, and they are not meeting the Dietary Guidelines for Americans [4, 5]. For example, the overall average dietary quality score for working-age Americans (19–59 years), as measured by Healthy Eating Index (HEI-2015), was 58 out of 100, with 100 indicating complete alignment with the 2015–2020 United States (U.S.) Dietary Guidelines for Americans [6]. In addition, according to data from the 2019 Behavioral Risk Factor Surveillance System (BRFSS), approximately 12% of adults in the U.S. met the 2020–2025 Dietary Guidelines for fruit intake, and 10% meet the guidelines for vegetables [7].

The workplace may play an important role in shaping the eating behaviors of U.S. adults, as many spend most of their day in the workplace environment. However, surveys of working adults in the U.S. have found that foods and beverages obtained in the workplace (either purchased or for free) tend to be low in nutritional quality and energy dense [8, 9]. In addition, workplace health promotion programs and healthier foods policies are limited, especially among worksites with smaller numbers of employees [10].

Unhealthy diets and associated chronic conditions have been linked to lost productivity, absenteeism, and higher healthcare costs for employers [11, 12]. As a result, employers have an interest in promoting healthy eating practices at the workplace and beyond to support the health of their workforce. The Centers for Disease Control and Prevention (CDC) and the World Health Organization (WHO) have also identified the workplace as an important location for health promotion [13, 14], and the CDC recommends that guidelines for foods and beverages served at worksites align with the most recent version of the U.S. Dietary Guidelines for Americans [15].

Several factors have been identified that may influence eating behaviors in the workplace, including the availability and cost of food, time available for eating, workplace stress or pressure, provision of nutrition information, and social norms or the influence of work colleagues [16–19]. More broadly, taste, healthfulness, convenience, and price have been found to be important determinants of meal choice across different populations [20, 21], but the relative importance of these factors has been found to differ by demographic characteristics [18, 21, 22]. For example, data from a study of U.S. working adults indicate that older workers view convenience and health as the most important factors influencing their meal choices, but younger workers reported convenience and taste as the most important factors [18]. Results from a survey of an Irish working population indicate food choice motives vary by sex, with females reporting to a greater extent than males that price, sensory appeal, health, and convenience were more important to their food choice [22].

While studies examining how factors influencing reported food choice in worksites vary by demographic characteristics are important, they are limited in the fact that they do not assess actual purchases. As a result, questions remain about the best way to approach the promotion of healthy food purchases among employees and to what extent health promotion activities should be tailored to the demographic characteristics of the employees at particular worksites.

The purpose of this study was to (1) assess the nutritional quality of lunchtime meal purchases by employees in cafeterias of a large organization, (2) examine associations between lunchtime meal quality selection and the demographic characteristics of employees, and (3) determine the healthfulness of foods and beverages offered in the cafeterias of this organization during data collection. Results from this study can be used to inform future interventions to promote healthy eating in workplaces.

## Methods

### Design

This cross-sectional study is a secondary analysis of data from the Effects of Physical Activity Calorie Expenditure (PACE) food labeling study implemented in three worksite cafeterias that are part of one large not-for-profit health insurance organization located in the Southeastern United States. Details about the design and results of the PACE study were published elsewhere [23, 24]. Briefly, PACE was a three-year quasi-experimental study (2015–2017) that compared the effects of PACE labels to calorie-only labels in worksite cafeterias on calories purchased before and after the label intervention. Results of the main study showed that the PACE labeling and calorie-only labeling interventions both resulted in a modest decrease in lunchtime calories purchased, but there were no significant differences between the two types of labels [24].

### Participants

There were 414 employees who participated in the original PACE study. Data from a subset of the original participants were included in this secondary analysis (*n* = 378). Participants were included if there was complete data for at least one meal purchased in one of the cafeterias during the original three-year study. No new participants were recruited for this analysis, and all data were collected between 2015 and 2017. The characteristics of the subsample (*n* = 378) are provided in Table 1 and were similar to those of the original sample (*n* = 414) and full employee population at the time of the study.

### Data collection

Demographic information and medical and dietary history were collected at baseline on an electronic tablet [23]. Participants self-reported whether they had a history of high blood pressure, high cholesterol, and/or diabetes. Participants also completed a health literacy assessment (Newest Vital Sign) [25] and a three-item numeracy assessment [26] at baseline. Adequate numeracy was defined as at least two out of three items correct, and health literacy was defined as at least five out of six items correct. Biometric data, including height, weight, and body mass index (BMI), were also collected [23]. To capture data on physical activity, participants completed a physical activity assessment form [27, 28], and were asked to wear an accelerometer (Actigraph wGT3X-BT) at two time points during the study (baseline and during the intervention year).

Data on meals purchased by all participants were collected using a photo capture system and notes from onsite study coordinators [23, 24]. Study coordinators helped participants place their food on a shelf and took a picture of the entire meal they had purchased. Meal photos were taken over 2-week periods every three months during the study. A list of menu items served across the three worksite cafeterias and serving sizes for these items were provided by the cafeteria staff. When serving sizes were not provided, they were estimated using a food atlas developed for the study [23]. Nutrient information for the menu items was compiled using U.S. Department of Agriculture nutrient databases, and the process is explained in detail elsewhere [29].

### Meal and menu quality

The nutritional quality of meals selected by participants and the menu items offered in the cafeterias were assessed using the Healthy Eating Index 2015 (HEI-2015). The HEI-2015 is an index designed to determine how well a set of foods aligns with the 2015–2020 U.S. Dietary Guidelines for Americans [6]. The total overall score for HEI-2015 ranges from 0 to 100, with a higher score indicating better alignment with the Dietary Guidelines [30]. The total score is based on 13 subscores that range from 0 to a maximum score of either 5 or 10 [30]. The 13 subscores measure the extent to which the Dietary Guidelines for Americans for whole grains, total vegetables, greens and beans, fruits (total and whole), dairy, total protein foods, seafood and plant proteins, and fatty acids are adequately met (adequacy components 1 through 9) and the extent to which refined grains, sodium, added sugars, and saturated fats are included in moderation (moderation components 10 through 13) [30]. A graded approach has been used to interpret HEI-2015 scores [30]. Overall scores of 90–100 represent an “A” grade, 80–89 a “B”, 70–79 a “C”, 60–69 a “D”, and 0–59 an “F.” Component or subscores can be interpreted in a similar way. For example, 90–100% of the maximum component score is an “A.”

An HEI-2015 total score and component scores were calculated for the entire menu of options available in all the cafeterias during the study period. The HEI-2015 total score and component scores for the menu items were generated using a SAS macro for the Population Ratio Method [31].

The HEI-2015 meal quality scores were generated by combining foods and beverages purchased by participants into “meals” using SAS software (version 9.43) [32]. A SAS macro (HEI-2015 scoring macro) provided by the National Cancer Institute was used to generate HEI-2015 component scores and an HEI-2015 total score for each of the meals purchased by each participant [31]. Additional details about the meal quality scores are described elsewhere [29]. The mean of the HEI-2015 total scores and component scores were then calculated for each participant.

### Data analysis

Summary statistics, such as means, standard deviation, range, and percentiles, were generated for the meal index. The analysis of variance (ANOVA) was used to compare the HEI-2015 scores between groups of a categorical variable. An equal variance assumption was assumed. However, if the assumption was rejected by Levene’s test, we used Welch’s robust test to test the difference between group means.

**Table 1:** Demographic characteristics of participants in the Effects of Physical Activity Calorie Expenditure (PACE) Food Labeling research study (2015–2017) (*n* = 378)

Age < 45, N (%)	219 (57.9)
Female, N (%)	295(78.0)
Race, N (%)	
White	174 (46.0)
Black	165 (43.7)
Asian	21 (5.6)
Other	18 (4.8)
Hispanic ethnicity, N (%)	18 (4.8)
Education level, N (%)	
High school	47 (12.4)
Technical school/Associates degree	86 (22.8)
College graduate	144 (38.1)
Master’s degree+	101 (26.7)
Current smoker, N (%)	19 (5.0)
Adequate^a^ numeracy level, N (%)	211 (55.8)
Adequate^b^ literacy level N (%)	257 (68.0)
Self-reported health status, N (%)	
Excellent/very good	207 (54.8)
Good	144 (38.1)
Fair/poor	27 (7.1)
Total yearly household income, N (%)	
$25,000-$49,999	116 (30.7)
$50,000-$99,999	137 (36.2)
$100,000+	125 (33.1)
Occupation description, N (%)	
Administration/clerical	68 (18.0)
Customer service/sales	86 (22.8)
Financial/technical	121 (32.0)
Management	101 (26.7)
Mean body mass index, kg/m^2^	
< 25	150 (39.7)
25.1–29.9	80 (21.2)
> 30	148 (39.2)
Hypertension, N (%)	92 (24.3)
High cholesterol, N (%)	91 (24.1)
Physical activity level, N (%) (minutes per week)	
1–0-59	55 (14.6)
2–60-149	108 (28.6)
3–150-299	60 (15.9)
4–300+	29 (7.7)

^a^2 or 3 correct out of 3 items

^b^5 correct out of 6 items

## Results

Of the original 414 PACE participants, 378 had data on at least one meal and complete demographic information and were included in the analyses. The number of meals per participant ranged from 1 to 67 (mean = 21.3, Standard Deviation (SD) = 15.9). The mean Healthy Eating Index-2015 (HEI-2015) score for lunch observations across participants was 47.1 (SD = 6.8) with a range of 15.0-74.7.

Table 2 provides an overview of the associations between the characteristics of participants and their average HEI-2015 total score (across all meals). Associations between participant characteristics and the HEI-2015 subcomponent scores are provided in an additional file [see Additional file 1]. The associations between HEI-2015 total meal scores and age, sex (self-disclosed), body mass index (BMI), and history of high blood pressure, high cholesterol, or diabetes were not statistically significant. Significant associations were found between meal quality and race, education level, current smoker status, numeracy level, health literacy level, self-reported health status, total yearly household income, occupation description, and physical activity level. By race, the average HEI-2015 scores were significantly higher for individuals who self-identified as Asian, followed by those who self-identified as other, White, and Black. A dose-response relationship was present for education – as education level increased, meal scores also increased. Current smokers had lower meal quality scores than participants who were not current smokers. Individuals with adequate scores on numeracy and individuals with adequate scores on health literacy had higher meal quality scores than those who did not have adequate scores. Meal quality scores increased as household income increased and as physical activity increased. Finally, individuals who worked in management or financial/technical jobs had higher meal quality scores than those who worked in administration/clerical or customer service/sales jobs.

**Table 2:** Associations between various demographic characteristics of participants in the Effects of Physical Activity Calorie Expenditure (PACE) Food Labeling research study (2015–2017) and their average HEI-2015 meal score (*n* = 378)

Characteristic	Healthy Eating Index-2015 Meal Score Mean (Standard Deviation)	*p*-value
Age group		0.09
Age < 45	46.6 (7.0)	
Age ≥ 45	47.8 (6.5)	
Sex		0.13
Female	46.7 (6.0)	
Male	48.3 (9.0)	
Race		< 0.001^*^
Asian	51.5 (9.0)	
Other	48.1 (5.4)	
White	47.8 (7.1)	
Black	45.7 (5.8)	
Education level		< 0.001^†^
High school	44.6 (5.8)	
Technical school/Associates degree	46.3 (6.6)	
College graduate	47.0 (6.5)	
Master’s degree+	49.1 (7.2)	
Current smoker		< 0.001
Yes	41.9 (7.3)	
No	47.4 (6.7)	
Adequate^a^ numeracy level		0.008
Yes	47.9 (6.8)	
No	46.0 (6.6)	
Adequate^b^ health literacy level		0.005
Yes	47.8 (6.4)	
No	45.6 (7.4)	
Self-reported health status		0.047^‡^
Excellent/very good	47.9 (7.2)	
Good	46.1 (6.5)	
Fair/poor	46.5 (4.6)	
Total yearly household income		< 0.001^§^
$25,000-$49,999	44.9 (6.1)	
$50,000-$99,999	47.1 (6.6)	
$100,000+	49.1 (7.0)	
Occupation description		0.006^¶^
Administration/clerical	46.0 (5.3)	
Customer service/sales	45.3 (7.3)	
Financial/technical	48.0 (6.6)	
Management	48.2 (7.2)	
Physical activity level (minutes per week)		< 0.001^#^
1–0-59	44.5 (5.4)	
2–60-149	47.7 (5.9)	
3–150-299	46.4 (7.2)	
4–300+	50.5 (6.0)	
Body mass index (kg/m2)		0.18
<25	47.9 (6.5)	
25.1–29.9	47.9 (7.2)	
≥30	46.5 (6.1)	
High blood pressure		0.86
Yes	47.2 (5.9)	
No	47.0 (7.0)	
High cholesterol		0.87
Yes	47.2 (7.5)	
No	47.1 (6.6)	
History diabetes		0.96
Yes	47.0 (6.6)	
No	47.1 (6.8)	

^a^ 2 or 3 correct out of 3 items

^b^ 5 correct out of 6 items

* Significant comparisons: White – Black; Black- Asian

^†^ Significant comparisons: High school – Master’s +; Tech school/Assoc. deg. – Master’s +

^‡^ Significant comparisons: Excellent/very good – Good

^§^ Significant comparisons: All pairwise

^¶^ Significant comparisons: Customer services/sales-Management; Financial/technical-Management

^#^ Significant comparisons: Physical activity level 1–2; 1–4; 3–4

The total mean HEI-2015 score for the menu items offered in the cafeteria during the study period was 63.1. Table 3 contains the mean component scores for the menu items.

**Table 3:** Cafeteria-level Healthy Eating Index-2015 and component scores computed from the 1,229 menu items offered during the data collection period (2015–2017) in the worksite cafeterias

	Maximum Score Possible	HEI-2015 Score Mean (Standard Error)	Percent of Maximum Score
Total HEI-2015 Score	100	63.1 (1.8)	63.1
**Adequacy**:			
Total Vegetables	5	3.9 (0.2)	78
Greens and Beans	5	3.8 (0.5)	76
Total Fruits	5	2.4 (0.2)	48
Whole Fruits	5	4.1 (0.3)	82
Whole Grains	10	4.5 (0.5)	45
Dairy	10	3.8 (0.3)	38
Total Protein Foods	5	4.9 (0.1)	98
Seafood and Plant Proteins	5	5.0 (0)	100
Fatty Acids	10	7.8 (0.8)	78
**Moderation**:			
Sodium	10	4.2 (0.6)	42
Refined Grains	10	6.6 (0.5)	66
Saturated Fats	10	5.3 (0.5)	53
Added Sugars	10	6.8 (0.3)	68

## Discussion

In this study, we found that the average nutritional quality of meals selected by study participants in the worksite cafeterias was 47.1 out of 100 – a failing grade on the grade scale that has been used to interpret HEI-2015 [30]. The highest score for an individual meal was 89.6. In addition, certain HEI-2015 meal subcomponent scores were below 50% of the maximum subcomponent score (greens and beans, total fruit, whole fruits, whole grains, dairy, seafood and plant protein, and sodium). Studies examining the nutritional quality of items obtained in the workplace are limited, but our findings are consistent with another study that assessed the nutritional quality of items obtained from the work environment [8]. Onufrak et al. [8] used the 2010 Healthy Eating Index (HEI-2010) to assess the dietary quality of foods that were purchased or obtained for free in the workplace and estimated an average score of 48.2 out of 100 and noted that items were especially low in total fruits and whole grains.

There are several possible reasons for the low meal quality scores found in this study. The foods and beverages available in the cafeteria and the prices of these items could have influenced the selection of items by participants. Price has been shown to influence food selection [16–18, 20, 21] and might be a particularly important factor in food choices made by lower-income consumers [17, 20]. The HEI-2015 score for all the menu items offered during the study period indicates that healthy options were available to participants, but several less healthy items were also available for purchase. The HEI-2015 score for all of the menu items in the cafeterias was approximately 63, and the component scores for total fruit, dairy, and items to moderate were between 38% and 68% of the maximum subcomponent scores, indicating that the menu items were not in alignment with the Dietary Guidelines for Americans. However, the HEI-2015 component scores for total vegetables, beans and greens, whole fruits, total protein foods, seafood and plant proteins, and fatty acids (the ratio of poly- and monounsaturated fat to saturated fat) were between 76% and 100% of the maximum subcomponent scores. It is important to note that the full menu of items analyzed for this study was not available at every cafeteria and on every observation day. If the healthier entrée items available on particular days did not look appealing to participants, it’s possible they may have selected less healthy quick service items. The availability of unhealthy items may also reflect a response to consumer demand, and/or the purchase of these items could have been due to price differences between healthy and less healthy options. Participant preferences and price differences are important to consider for future research.

Another reason for the lower average meal scores could have been the result of using HEI-2015 as the index for measuring meal quality. The HEI-2015 was designed to assess overall diet quality and the nutritional quality of food supplies [33, 34]. To measure overall diet quality, HEI-2015 scores are often based on multiple eating occasions over the course of a day or several days. For this study, HEI-2015 scores were generated from meal observations, which may not reflect participants’ overall dietary patterns. It is possible that individuals were consuming various food groups at different eating occasions and thus did not select these components at lunch. As a result, HEI-2015 meal scores should not be expected to be “perfect” or reach the maximum score possible.

Nevertheless, it is also possible that the meal scores may be associated with or reflect the overall dietary patterns of participants. Results from a study of the U.S. workforce that compared the nutritional quality of items obtained by study participants at workplaces to overall HEI-2015 dietary quality scores indicate that participants who purchase or obtain healthier items at work tended to have higher overall dietary quality scores [9]. Individuals with healthier purchases at work also tended to have a lower prevalence of cardiometabolic risk factors (obesity, hypertension, prediabetes/diabetes, and/or hyperlipidemia) compared to individuals with the least healthy purchases [9]. In addition, according to 2017–2018 NHANES data, working-age Americans (19–59) have an average overall HEI-2015 total score of 58 and lower HEI-2015 subcomponent scores for fruit and whole grains [6]. While this overall diet quality score is higher than the average score for meals in this study, it still indicates that the diet and meal quality of working-age Americans needs to improve.

In this study, we also found significant associations between average meal quality scores and race, education level, smoking status, income, physical activity levels, occupation type, and health literacy and numeracy scores. Studies examining associations between demographic characteristics and worksite meal quality or worksite food purchases are limited. However, findings from this study were consistent with some findings from a study that assessed demographic differences in meal quality from full-service and fast-food restaurants among NHANES participants [35]. For that study, researchers used the American Heart Association (AHA) diet score as a measure of meal quality and found, similar to this study, that meal quality from full-service restaurants tended to be lower among non-Hispanic Blacks and individuals with lower education levels. Unlike this study, meal scores from fast food and full-service restaurants tended to also be lower among adults with overweight or obesity [35].

Some similar trends have also been observed in studies that have examined associations between demographic characteristics and scores from indices that measure overall diet quality. Similar to this study, diet quality scores have been found to be higher among non-Hispanic Asians compared to non-Hispanic Whites and non-Hispanic African Americans [36, 37]. In addition, studies have found that individuals who have higher dietary quality scores are more likely to have higher incomes [37–39] and higher education levels [37, 39–42]. Smoking status has also been linked to overall diet quality. Individuals who have never smoked tend to have a better diet quality than former smokers and current smokers [39, 42, 43]. Furthermore, individuals with higher dietary quality scores also tend to be more physically active than those with lower scores [39, 42, 44].

We did not find significant differences in meal scores by age, sex, self-reported health status, body mass index, or history of high blood pressure, high cholesterol, or diabetes status. However, significant differences in meal and diet quality scores have been reported in previous studies by sex, with women tending to have higher scores than men [35, 39, 41]. Older populations have also been found to have higher meal and dietary quality scores [35, 41, 42]. Studies have also found an inverse relationship between obesity and dietary indices [45].

The demographic differences found in meal quality selection in this study and in studies of overall diet quality warrant further investigation, as do potential interventions to address possible disparities in knowledge about nutrition or access and availability of healthier foods that may exist. For example, efforts to address dietary disparities by education level could be addressed through interventions that focus on increasing consumers’ knowledge about nutrition through tailored communication or providing more information at the point of purchase about the healthfulness of items [46–48]. Pricing strategies could also be employed to decrease the cost of healthier food and make it more accessible to people with lower incomes [49, 50]. However, while there were significant differences in meal quality scores between various demographic groups in this study, overall, these differences were not large. The results indicate that there is considerable room for improvement in healthy meal selection among most participants.

As mentioned previously, workplace interventions may be an important area of focus for improving health behaviors overall and for decreasing disparities in diet and meal quality among various populations in the U.S. For example, multicomponent workplace interventions that focus on improving food quality, reducing portion sizes, increasing employee’s knowledge of and/or motivation for purchasing healthy food, reducing the price of healthy foods, and/or targeting food choice at the point of purchase (e.g. labeling or signage for healthier options) have been found to be effective at improving eating behaviors [49, 50] and in some cases body weight and cardiometabolic risk factors [50]. In addition, many studies focused on changing the food environment to promote healthy eating in workplaces have resulted in a decrease in the number of calories purchased, an increase in sales of healthier options, and/or an improvement in fruit and vegetable consumption [51, 52]. Implementing behavioral design strategies, sometimes referred to as choice architecture, may help point people toward healthier options and be effective in changing behaviors when it comes to making food choices [53–55]. Finally, implementing food guidelines or policies like the U.S. Federal Food Service Guidelines (FFSG) in government and private workplaces may help increase healthy options for employees, improve health outcomes, and decrease healthcare costs [56].

### Limitations

The purpose of this study was to examine possible differences in meal quality by demographic characteristics and not necessarily the factors contributing to differences in the selection of food in the workplace environment. Given the observational study design, we cannot conclude that there is a causal relationship between demographic characteristics included in this study and food selection. There may be other factors, beyond the ones described here, influencing food choice that could be addressed in future workplace food environment interventions. For example, the price and placement of healthy and less healthy items may have driven the selection of items in the cafeteria. It is possible that less healthy items were available at lower prices than healthier items, and less healthy items were more prominently displayed in the cafeterias. The prices of the menu items and menu item placement within the cafeteria were not collected as part of this study.

It is also important to note that the demographic categorizations used for this study are imprecise measures and likely do not capture the heterogeneity of individuals in these groups. For example, race is a social construction that does not have a biological basis [57–59], and the definitions of races have changed over time [60]. Race, however, has been described as a proxy measure for historic and ongoing discrimination and systematic racism experienced by marginalized racial groups, and racism can impact health behaviors and result in health disparities [57, 61, 62]. Along these lines, one potential mechanism that could explain some of the differences in food selection by race is exposure to food marketing. Studies have shown that African Americans are disproportionately targeted and exposed to advertisements for energy-dense and low-nutritional quality foods and beverages [63–67], which may influence food preferences and consumption [65, 68].Another potential limitation, as previously mentioned, is the use of HEI-2015 as the index for measuring meal quality may have contributed to the lower average meal scores. As far as we are aware, a gold standard index for measuring meal quality in the U.S. population does not exist. Despite this, scores for individual meals using HEI-2015 may provide valuable information for consumers, public health and nutrition professionals, and researchers. HEI-2015 and tools like the USDA’s MyPlate may serve as starting points for creating an index designed to assess meal quality. For example, the 2020–2025 Dietary Guidelines suggest adding fruit to meals may be a good way to achieve the recommended amount for the day, and MyPlate provides a visual for what a single meal should contain to meet the dietary guidelines [5].

## Conclusion

While we found significant associations between average lunchtime meal quality scores and several important demographic characteristics, we also found low meal scores across most study participants. In addition, the overall HEI-2015 score for menu items offered in the three cafeterias during the data collection period was moderate, but healthier options were available. These results suggest that healthy eating promotion activities in workplaces may need to be tailored to the demographic characteristics of the employees, and efforts to improve the food environment and promote health in the workplace could improve meal quality for all employees.

### Electronic supplementary material

Below is the link to the electronic supplementary material.

Supplementary Material 1

## Data Availability

The datasets generated and/or analyzed during the current study are not publicly available due to privacy or ethical restrictions but are available from the corresponding author on reasonable request.
